# Enhanced post-exercise hypotension in female athletes with menstrual disorders

**DOI:** 10.1016/j.jsampl.2026.100137

**Published:** 2026-03-05

**Authors:** Mariko Nakamura, Yoko Saito, Kazumi Eguchi, Koichiro Hayashi

**Affiliations:** a Japan Institute of Sports Sciences, Department of Sports Sciences and Research, Tokyo, Japan; b Toyama Prefectural University, Faculty of Engineering, Toyama, Japan; c Nippon Sport Science University, Tokyo, Japan; d Kokugakuin University, Department of Human Development, Yokohama, Japan

**Keywords:** Amenorrhea, Blood pressure, Cardiovascular function, Hypoestrogenism, Recovery

## Abstract

**Background:**

To investigate whether the magnitude of post-exercise hypotension (PEH) is greater in female endurance athletes with oligomenorrhea/amenorrhea (OAA) compared with eumenorrheic (EA) athletes, and to examine the associated hemodynamic correlates.

**Methods:**

Ten EA athletes (tested during the early follicular phase) and eight OAA athletes performed incremental cycle exercise to exhaustion. Brachial blood pressure (BP), cardiac output (CO), total peripheral resistance (TPR), and spontaneous baroreflex sensitivity (SBRS) were assessed at baseline and at 15 (P15), 30 (P30), and 60 (P60) min post-exercise in the supine position. Linear mixed-effects models were used to compare temporal responses between groups.

**Results:**

Both groups demonstrated significant PEH. Absolute systolic BP showed no between-group differences at baseline or during post-exercise recovery, whereas absolute diastolic BP (DBP) and mean arterial pressure (MAP) were higher at baseline in the OAA group. When expressed as changes from baseline, reductions in DBP and MAP at P60 were significantly greater in the OAA group than in the EA group (both *p* < 0.05). Post-exercise changes in CO and relative changes in TPR and SBRS demonstrated significant main effects of time (*p* < 0.05) but showed no significant between-group differences.

**Conclusions:**

These findings suggest that chronic hypoestrogenism may augment PEH in female endurance athletes through mechanisms not fully explained by systemic TPR or SBRS.

## 1 Introduction

Post-exercise hypotension (PEH) – a consistent fall in arterial blood pressure (BP) following a single session of aerobic exercise [1] – is well described. However, the magnitude of PEH is highly context-dependent, influenced significantly by factors such as exercise intensity and recovery posture [2,3]. Although mechanisms remain incompletely defined, PEH has been attributed to an altered baroreflex function [4], sympathetic withdrawal leading to persistent vasodilation, and transient reductions in cardiac output (CO) [5].

Findings regarding sex differences in PEH remain inconclusive. In trained women, the contribution of vasodilatory mechanisms may be relatively prominent [6]. Across the normal menstrual cycle, PEH tends to be attenuated in the late-follicular and mid-luteal phases –when ovarian hormone concentrations are higher–than in the early-follicular phase, when ovarian hormone concentrations are low [7]. These patterns are consistent with the hypothesis that high ovarian hormones may provide autonomic “buffering” against post-exercise BP fluctuations.

Menstrual disturbances are prevalent among endurance athletes [8]; those with oligomenorrhea or amenorrhea (OAA) typically are characterized by a chronic hypoestrogenic state. Given that PEH varies across menstrual phase in eumenorrheic (EA) women, the absence of cyclic hormonal fluctuations suggests that athletes with menstrual dysfunction may exhibit distinct cardiovascular recoveries. Importantly, excessive PEH may increase the risk of reduced cerebral perfusion and post-exercise syncope [5]. For endurance athletes, ensuring safety during the recovery period following intense training is a practical concern, as exercise-induced hypotension can lead to light-headedness and an increased risk of falls. However, data characterizing PEH and its hemodynamic correlates in this population remain sparse. Therefore, we compared the temporal recovery of BP and associated cardiovascular responses – CO, total peripheral resistance (TPR), and spontaneous baroreflex sensitivity (SBRS) – after maximal exercise in EA vs OAA athletes. We hypothesized that OAA athletes would exhibit a greater magnitude of PEH compared to EA athletes.

## 2 Method

### 2.1 Participants

Eighteen healthy female endurance athletes were classified by menstrual status: eumenorrheic (EA; n = 10, 18.9 ± 0.4 y, 158.8 ± 3.7 cm, 49.4 ± 3.3 kg) and oligomenorrheic/amenorrheic (OAA; n = 8, 18.9 ± 0.6 y, 161.2 ± 4.2 cm, 52.0 ± 7.8 kg). Of the eight OAA participants, the two with oligomenorrhea (>75-day cycles) progressed to amenorrhea (>90 days) after the study. Participants' menstrual status was verified based on the framework of Elliott-Sale et al., [9] using a detailed menstrual history questionnaire and at least three months of daily basal body temperature (BBT) records. All were intercollegiate runners (6.0 ± 0.2 d wk^−1^; 3.1 ± 0.2 h d^−1^), normotensive, non-smokers, medication-free. Written consent was obtained; ethics approval: Japan Institute of Sports Sciences (2014-347), in accordance with the Declaration of Helsinki.

### 2.2 Procedures and exercise protocol

EA were assessed in the early-follicular phase, (4–8 days after the onset of menstruation [9]); OAA were tested on a non-phased day. Participants arrived after a 12-h fast and ≥16 h exercise abstention; sessions were scheduled at a consistent time of day. To avoid potential diurnal variations, participants were tested at the same time of day throughout the study period (6:00–10:00 am). After a 20-min supine rest, baseline measures were obtained, followed by an incremental cycling test: a 5-min warm-up at 1.0 kp, then an increase of 0.5 kp every 3 min to exhaustion using a cycle ergometer (Power Max VII; Konami Sports Co., Ltd., Tokyo, Japan). Although exercise duration varied individually, this protocol ensured that all participants reached a comparable relative physiological stress prior to the recovery phase. Breath-by-breath gas exchange (AE300S, Minato Medical Science Co., Ltd., Osaka, Japan) yielded V̇O_2__max_ and V̇E _max_. Maximal effort criteria included V̇O_2_ plateau and/or respiratory exchange ratio >1.10, rate of perceived exertion >17, and absent HR rise. Post-exercise assessments were repeated supine at 15 (P15), 30 (P30), and 60 (P60) min.

### 2.3 Measurement of cardiovascular parameters

Brachial systolic BP (SBP), diastolic BP (DBP), and mean arterial pressure (MAP) were measured beat-to-beat using a continuous blood pressure monitor via applanation tonometry at the radial artery (Jentow-7700; Nihon Colin Co., Ltd., Komaki, Japan). This device non-invasively captures arterial pressure waveforms to provide beat-to-beat data. Echocardiography (SSD-6500, Aloka Co., Ltd., Tokyo, Japan) assessments were conducted in accordance with the protocol described by Saito et al. [10] and provided left ventricular internal dimension in diastole (LVIDd)/left ventricular internal dimension in systole (LVIDs); LV volumes were calculated by the Teichholz method [11] to derive stroke volume (SV = Left ventricular end-diastolic volume (LVEDV) – left ventricular end-systolic volume (LVESV)), ejection fraction (EF), cardiac output (CO = HR × SV), and total peripheral resistance (TPR = MAP/CO). Between-day coefficient of variation for SV was 6.8%. SBRS was determined by the sequence method during a 5-min supine recording with paced breathing (0.25 Hz). R–R intervals and SBP were recorded using wrist arterial tonometry (Jentow-7700) and electrocardiography, with data sampled at 1000 Hz. Baroreflex sequences, defined by a slope of ≥3 consecutive beats with an SBP change ≥1 mmHg and an R–R interval change ≥1 ms (lag 1) were identified. Sequences with R^2^ > 0.85 were averaged to calculate spontaneous cardiovagal baroreflex sensitivity [12]. These cardiovascular parameters were measured at baseline and 15, 30, and 60 min after exercise.

### 2.4 Blood sample

A 10-ml venous blood sample was collected from the antecubital vein of each participant, centrifuged at 3000 rpm for 10 min, and the serum was stored at −80 °C until analysis. Serum concentrations of luteinizing hormone (LH), follicle-stimulating hormone (FSH), estradiol, and progesterone were determined using radioimmunoassay.

### 2.5 Statistical analysis

All statistical analyses were performed using R statistical software (R Core Team, Vienna, Austria). Data are presented as means ± standard deviations (SD). To compare the time-course changes in hemodynamic variables between groups of blood pressure, linear mixed-effects models (LMM) were employed using the lme4 and lmerTest packages. The model included “Group” (EA vs. OAA), “Time” (baseline, P15, P30, and P60), and their interaction (Group × Time) as fixed effects, and “Participant” as a random effect (random intercept) to account for repeated measures within subjects. When a significant interaction was observed, *post-hoc* pairwise comparisons were performed to identify differences between groups at each time point using the Bonferroni correction (implemented via the emmeans package). Participant characteristics were compared by unpaired t-test. Significance was set at *p* < 0.05.

## 3 Results

### 3.1 Participant characteristics

The EA and OAA groups were comparable in anthropometric parameters, V̇O_2max_, V̇E_max_, and serum concentrations of LH, FSH, estradiol, and progesterone concentrations, respectively (all *p* > 0.05; Table 1).

**Table 1:** Physiological characteristics and hormonal concentration.

		EA group	OAA group
Age	yrs	18.2 ± 0.4	18.9 ± 0.6
Height	cm	158.8 ± 3.7	161.2 ± 4.2
Body Mass	kg	49.4 ± 3.3	52.0 ± 7.8
BMI	kg/m^2^	19.6 ± 1.1	20.1 ± 2.8
V̇O_2max_	mL/kg/min	59.4 ± 5.7	56.8 ± 6.7
V̇E_max_	L/min	95.1 ± 11.9	93.8 ± 14.9
LH	mIU/mL	6.2 ± 3.8	4.3 ± 2.3
FSH	mIU/mL	4.3 ± 1.3	5.5 ± 3.0
Estradiol	pg/mL	25.4 ± 11.1	26.1 ± 12.4
Progesterone	ng/mL	0.9 ± 0.3	0.8 ± 0.1

Data are mean ± SD. EA, eumenorrheic athletes; OAA, oligomenorrheic or amenorrheic athletes; BMI, body mass index; V̇O_2max_, maximal oxygen consumption; V̇E_max_, maximal ventilation; LH, luteinizing hormone; FSH, follicle-stimulating hormone.

### 3.2 Blood pressure

Absolute SBP did not differ significantly between groups at baseline or during recovery; however, a trend toward a group × time interaction was observed (*p* = 0.05), with a greater post-exercise reduction in the OAA group. In contrast, absolute DBP and MAP exhibited significant group × time interactions (*p* = 0.017 and *p* = 0.047, respectively), with higher values observed in the OAA group at baseline; additionally, a between-group difference was evident for DBP at P30 (Table 2).

**Table 2:** Resting and post-exercise hemodynamic changes.

Variables		EA group				OAA group				*p* value		
		Baseline	15min	30min	60min	Baseline	15min	30min	60min	Group	Time	Interaction
HR	bpm	45 ± 7	70 ± 6	59 ± 5	53 ± 5	45 ± 7	70 ± 11	60 ± 12	54 ± 11	0.65	<0.01	0.96
SBP	mmHg	101 ± 6	104 ± 4	100 ± 6	98 ± 6	105 ± 5	105 ± 5	103 ± 6	99 ± 4	0.37	<0.01	0.05
DBP	mmHg	55 ± 3	53 ± 3	52 ± 4	53 ± 3	58 ± 4†	55 ± 3	55 ± 3†	54 ± 4	0.05	<0.01	<0.05
MAP	mmHg	70 ± 4	70 ± 3	68 ± 4	68 ± 3	74 ± 4†	72 ± 3	71 ± 2	69 ± 4	0.09	<0.01	<0.05
SV	ml	63.3 ± 8.0	59.4 ± 10.1	62.8 ± 11.4	61.7 ± 10.3	65.7 ± 10.0	57.1 ± 6.4	61.3 ± 8.1	64.7 ± 7.8	0.76	<0.01	0.22
CO	L/min	2.8 ± 0.5	3.7 ± 0.8	3.2 ± 0.5	3.1 ± 0.5	2.7 ± 0.6	3.7 ± 0.7	3.3 ± 0.5	3.2 ± 0.6	0.90	<0.01	0.89
LVIDd	cm	4.7 ± 0.3	4.6 ± 0.4	4.7 ± 0.3	4.7 ± 0.4	4.8 ± 0.2	4.7 ± 0.3	4.7 ± 0.2	4.8 ± 0.2	0.57	<0.05	0.51
LVIDs	cm	3.2 ± 0.3	3.1 ± 0.3	3.2 ± 0.2	3.2 ± 0.3	3.2 ± 0.3	3.3 ± 0.3	3.2 ± 0.3	3.2 ± 0.3	0.61	0.67	0.30
LVEDV	cm^3^	103.2 ± 15.6	98.8 ± 18.3	103.6 ± 17.1	102.2 ± 17.1	106.8 ± 12.2	100.9 ± 13.3	103.0 ± 11.6	107.2 ± 11.0	0.63	<0.01	0.33
LVESV	cm^3^	39.7 ± 8.9	39.4 ± 9.7	40.7 ± 7.4	40.2 ± 8.3	40.9 ± 8.2	43.6 ± 8.6	41.7 ± 8.1	42.5 ± 8.1	0.60	0.60	0.29
EF	%	61.7 ± 7.2	60.4 ± 7.6	60.6 ± 3.6	60.6 ± 3.6	64.5 ± 6.7	59.4 ± 4.0	59.6 ± 5.7	60.3 ± 5.7	0.78	<0.05	0.42
TPR	mmHg/L/min	26.2 ± 5.8	19.5 ± 3.9	21.4 ± 4.0	22.5 ± 3.8	28.4 ± 6.1	20.0 ± 5.0	22.2 ± 3.2	21.9 ± 3.7	0.56	<0.05	0.57
SBRS	mmHg/sec	40.9 ± 16.5	12.6 ± 5.4	25.6 ± 10.3	31.4 ± 16.5	48.0 ± 22.7	12.8 ± 9.2	23.5 ± 14.1	33.5 ± 18.5	0.48	<0.01	0.72

Data are mean ± SD. EA, eumenorrheic athletes; OAA, oligomenorrheic or amenorrheic athletes; HR, heart rate; SBP, systolic blood pressure; DBP, diastolic blood pressure; MAP, mean arterial pressure; SV, stroke volume; CO, cardiac output; LVIDd, left ventricular internal dimension in diastole; LVIDs, left ventricular internal dimension in systolic; LVEDV, left ventricular end diastolic volume; LVESV, left ventricular end systolic volume; EF, ejection fraction; TPR, total peripheral resistance; SBRS, spontaneous baroreflex sensitivity. †, *p* < 0.05 vs EA group at the same time point.

**Fig. 1:**
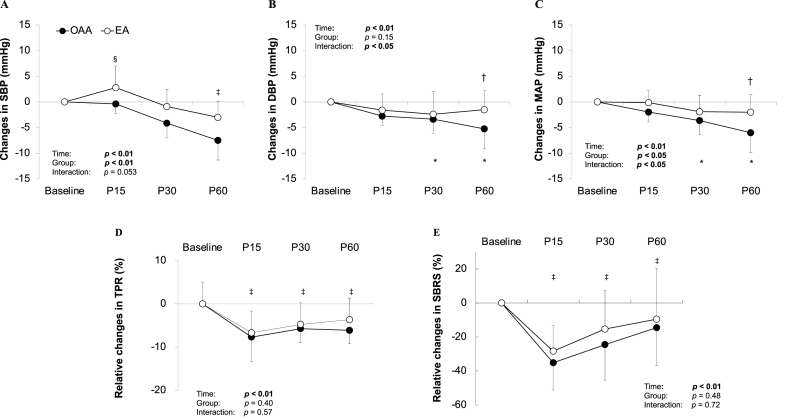
**Changes in BP and relative changes in TPR, and SBRS during post-exercise recovery.** Data are mean ± SD. SBP, systolic blood pressure; DBP, diastolic blood pressure; MAP, mean arterial pressure; TPR, total peripheral resistance; SBRS, spontaneous baroreflex sensitivity; EA, eumenorrheic athletes; OAA, oligomenorrheic or amenorrheic athletes. White circle is EA and black circle is OAA. A) Changes in SBP from baseline. SBP decreased in both groups, with a trend toward a group × time interaction (*p* = 0.053). B) Changes in DBP from baseline. Significant interaction (*p* < 0.05) was observed, where the reduction in DBP was significantly greater in OAA than in EA at P60. C) Changes in MAP from baseline. Significant interaction (*p* < 0.05) was observed, where the reduction in MAP was significantly greater in OAA than in EA at P60. D) Relative changes in TPR from baseline. TPR was also significantly reduced in both groups (*p* < 0.01, main effect of time), with no significant group differences. E) Relative changes in SBRS from baseline. SBRS was also significantly reduced in both groups (*p* < 0.01, main effect of time). §, p < 0.05 vs P60 based on the main effect of time; ‡, p < 0.05 vs baseline based on the main effect of time; ∗, p < 0.05 vs baseline within OAA group; †, p < 0.05 vs between groups at the same time point.

When analyzed as changes from baseline, significant group × time interactions were observed for DBP (*p* < 0.05; Fig. 1B) and MAP (*p* < 0.05; Fig. 1C). Post-hoc analysis revealed that the magnitude of the reduction at P60 was significantly greater in the OAA group compared to the EA group for both DBP (−5.3 ± 3.7 vs −1.5 ± 2.2 mmHg; *p* < 0.05) and MAP (−6.0 ± 3.4 vs −2.0 ± 2.1 mmHg; *p* < 0.05), indicating a more pronounced PEH response in OAA athletes. For SBP, a trend toward a group × time interaction was observed (*p* = 0.053; Fig. 1A).

### 3.3 Central and peripheral hemodynamic variables

Echocardiographic indices exhibited post-exercise changes but did not differ significantly between groups (Table 2). CO increased significantly during recovery period (main effect of time: *p* < 0.05, Table 2). Absolute values of TPR and SBRS decreased significantly during post-exercise recovery in both groups (main effect of time: *p* < 0.05), with no significant group differences or interactions (Table 2). Consistent with these findings, no significant group × time interactions were found for the relative changes in systemic parameters, including TPR (Fig. 1D) and SBRS (Fig. 1E). While both groups exhibited significant reductions in TPR and SBRS during recovery (main effect of time, *p* < 0.01), there were no significant between-group differences.

## 4 Discussion

We demonstrated cardiovascular recovery after maximal cycling in endurance athletes with menstrual disorders versus those with eumenorrhea. Both groups exhibited PEH, but the reduction, particularly in DBP and MAP, was significantly greater in the OAA group and persisted through 60 min of recovery. This augmented PEH was not accompanied by between-group differences in CO, SBRS, or TPR**,** suggesting that these systemic hemodynamic factors do not fully explain the observed group difference. To our knowledge, this is the first report showing greater PEH in athletes with chronic hypoestrogenism, highlighting menstrual status as a potential determinant of post-exercise blood pressure regulation.

The greater MAP reduction in OAA suggests that PEH was more effectively buffered in EA. Previous studies have shown that PEH is smaller in the mid-luteal than in the early-follicular phase [7], an attenuation commonly attributed to higher sympathetic activity and enhanced baroreflex function when ovarian hormones are elevated [13]. Because eumenorrheic athletes experience periodic rises in estrogen and progesterone, whereas cumulative estrogen exposure is lower in amenorrhoeic athletes [14], EA may retain cyclic autonomic buffering that is diminished in OAA. Given the importance of MAP for cerebral perfusion and orthostatic tolerance [15], the larger MAP fall at 60 min in OAA could increase susceptibility to post-exercise light-headedness, arguing for tailored recovery strategies in such athletes.

Consistent with our hypothesis, the magnitude of PEH in OAA was greater than in EA; however, these differences could not be attributed to post-exercise alterations in SBRS or TPR. In the present study, the OAA group exhibited statistically higher MAP and DBP compared to the EA group at rest. This observation is somewhat consistent with previous reports suggesting that amenorrheic athletes may have higher resting vascular resistance [14,16,17]; however, it is important to note that the blood pressure values for both groups in the present study remained well within the normal healthy range. Furthermore, because systemic TPR responses did not differ between groups during recovery, the more pronounced PEH in OAA may be driven by regional factors. One possible interpretation is that regional vasodilation in exercised limbs may contribute disproportionately to MAP reduction without substantially altering calculated systemic TPR. Consistent with this, previous reports in amenorrhoeic athletes show higher post-exercise calf blood flow and lower calf vascular resistance [14], implying limb-specific vasodilation. Although our protocol quantified systemic TPR, it did not directly measure limb blood flow; thus, localized vasodilatory contributions could be underestimated.

SBRS was suppressed after exercise in both groups through 60 min, consistent with typical cardiovagal withdrawal during recovery. Although estrogen [12,18] and regular aerobic exercise [19] can enhance cardiovagal activity, amenorrhoeic athletes may exhibit compensatory adaptations that preserve autonomic function: Wenner et al. found maintained autonomic and orthostatic responses in amenorrhoeic athletes [20]. Training can also strengthen central autonomic pathways [21] and baroreflex function [14], potentially offsetting adverse effects of low estrogen. In line with this, SBRS recovery and CO were comparable between groups, and LVIDd remained stable, suggesting preserved venous return and central hemodynamics irrespective of menstrual status. Collectively, these findings imply that central recovery is largely intact, whereas peripheral mechanisms (regional vasodilation and/or sympathetic withdrawal) more likely drive the larger relative BP fall in OAA.

These findings suggest that chronic hypoestrogenism may impair autonomic and peripheral vascular buffering, potentially predisposing athletes with menstrual disorders to altered cardiovascular recovery. From clinical and coaching perspectives, this necessitates individualized recovery protocols beyond simple monitoring, such as prolonged active cool-downs or intentional postural adjustments, to mitigate risks of symptomatic hypotension and post-exercise syncope. Given the prevalence of menstrual dysfunction in endurance sports, practitioners should integrate menstrual status into routine cardiovascular screenings to identify athletes at risk. Overall, our results underscore the importance of sex-specific, hormone-aware strategies to ensure safety and optimize recovery for female athletes.

## 5 Limitations

First, not all OAA met strict secondary amenorrhea criteria; however, two oligomenorrheic athletes included in this group remained anovulatory during study period and later developed amenorrhea, reflecting a state of prolonged hypoestrogenism. Second, EA were tested only in the early-follicular phase, precluding comparisons across different phases of the menstrual cycle. Third, MSNA was not measured; sympathoinhibition contributes to PEH [22,23], and sympathetic activity and BRS rise mid-luteal [13]. Whether OAA show altered post-exercise sympathoinhibition is unknown; future work should include limb blood flow, MSNA, and venous pooling to better elucidate the underlying mechanisms. Finally, exercise duration was determined by time-to-exhaustion rather than a fixed time interval. While this approach results in variation in absolute workload, it was intended to elicit maximal physiological stress in each participant prior to recovery measurements.

## 6 Conclusions

In the present study, OAA exhibited a more pronounced PEH compared to EA. While these differences were not explained by systemic cardiovascular parameters such as CO, TPR, and SBRS, they may be related to altered regional vascular responsiveness or localized vasodilatory mechanisms. This potentially predisposes female endurance athletes with menstrual disorders to greater post-exercise blood pressure reductions and reduced orthostatic tolerance. Further research is needed to elucidate the underlying regional or localized mechanisms.

**Key points**

**What is novel:** This is the first study to demonstrate that female endurance athletes with OAA exhibit a greater magnitude of PEH compared with eumenorrheic athletes following maximal exercise.

**What was found**: The exaggerated reduction in MAP in OAA athletes was not accompanied by differences in systemic TPR, CO, or SBRS during recovery, indicating that conventional systemic hemodynamic indices do not fully explain the observed group differences.

**Clinical implication**: These findings suggest that chronic hypoestrogenism may be associated with impaired autonomic and/or peripheral vascular buffering of post-exercise blood pressure, potentially predisposing female endurance athletes with menstrual disorders to altered cardiovascular recovery and reduced orthostatic tolerance following intense exercise.

## CRediT author statement

All authors contributed to the study conception. M.N., Y. S., K. E. have given substantial contributions to acquisition. M.N., Y. S., K. H. have critically contributed analysis and interpretation of the data. M. N. drafted the original manuscript, and the other authors critically reviewed and revised the manuscript draft. All authors approved the final version for submission.

## Funding

This study was funded by Grant-in-Aid for Scientific Research No. 26750318 from the 10.13039/501100001691Japan Society for the Promotion of Science.

## Declaration of competing interest

The authors declare that the research was conducted in the absence of any commercial or financial relationships that could be construed as a potential conflict of interest.
